# Supplementary material for: Human Papillomavirus 16 E6 Suppresses Transporter Associated with Antigen-Processing Complex in Human Tongue Keratinocyte Cells by Activating Lymphotoxin Pathway
Source: Cancers (Basel). 2022 Apr 12;14(8):1944. doi: 10.3390/cancers14081944 (PMC9028769; doi:10.3390/cancers14081944)

Supplementary Figure S1: **The expression of genes involved in MHC-I dependent antigen presentation in HNSCC cohort.** (**A** and **B**) The expression of genes involved in MHC-I heavy chain (A), and other genes MHC-I dependent antigen presentation (B) for OSCC and normal tissues. *** $p \leq 0.001$; ns—not significant.

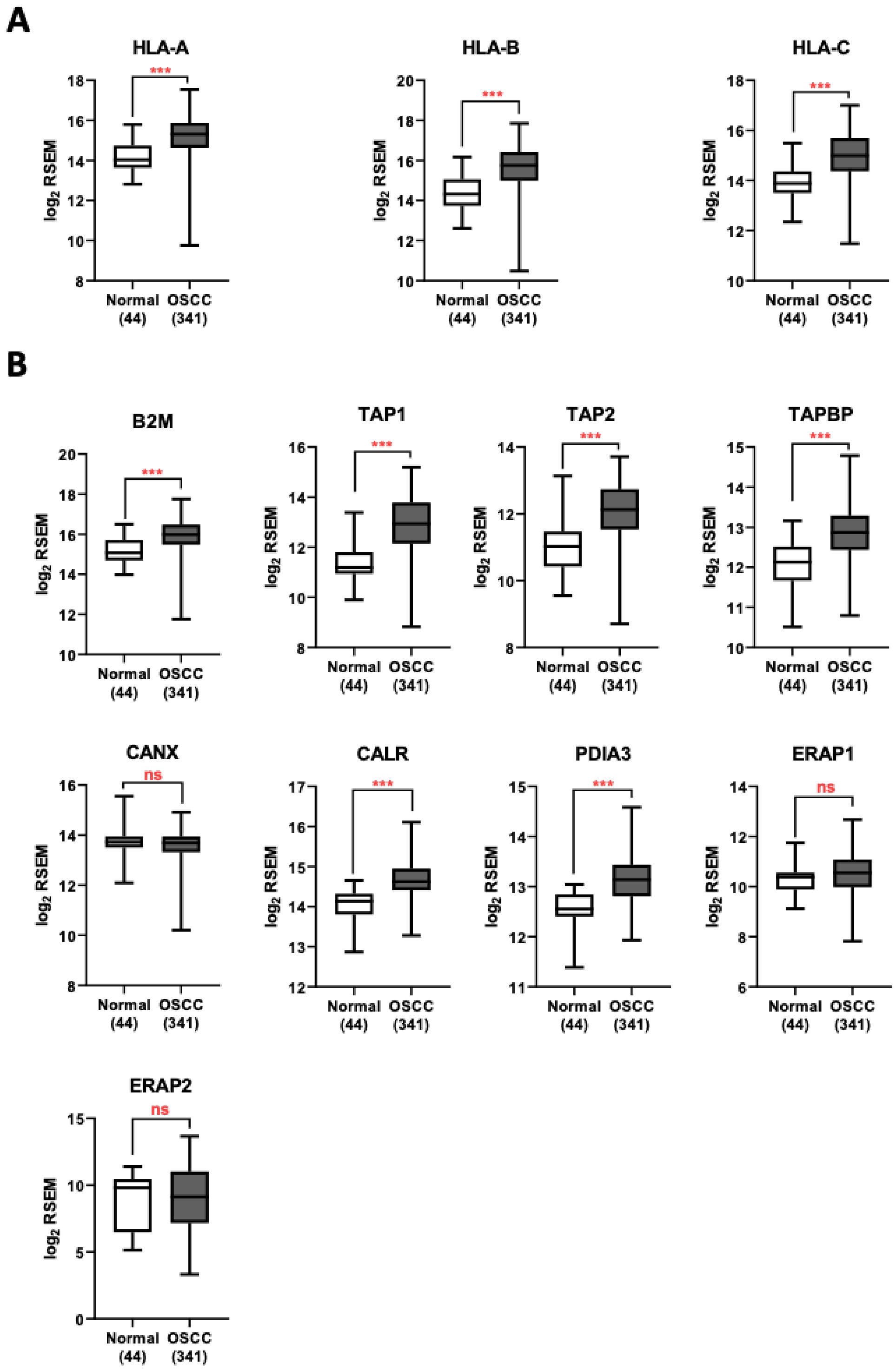

Supplementary Figure S2: **The expression of HPV16E6 in HTK1-K4DT and SCC90.** (**A**) The level of HPV16E6 after lentiviral transduction in HTK1-K4DT. (**B**) The efficiency of HPV16E6-specific siRNA measured by qRT-PCR. All experiments were carried out in triplicate, and at least three independent experiments were performed. * $p \leq 0.05$; ** $p \leq 0.01$; *** $p \leq 0.001$; ns—not significant.

A

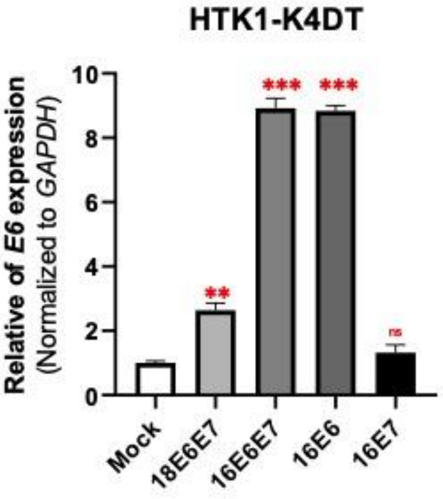

B

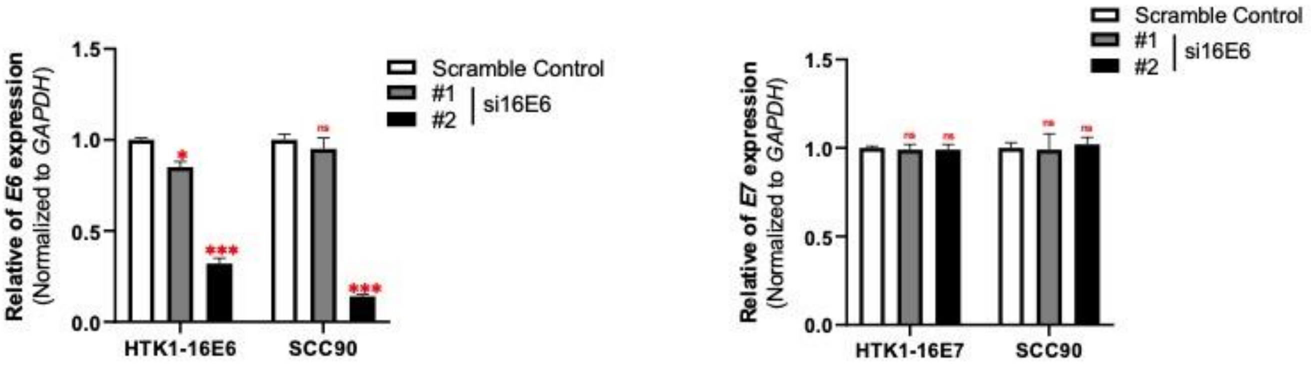

Supplementary Figure S3: **Original western blotting**. The level of indicated protein expression in (**A**) Figure 1D, **(B**) Figure 3B, (**C**) Figure 3E, (**D**) Figure 5B and 5D, (**E**). Figure 5G.

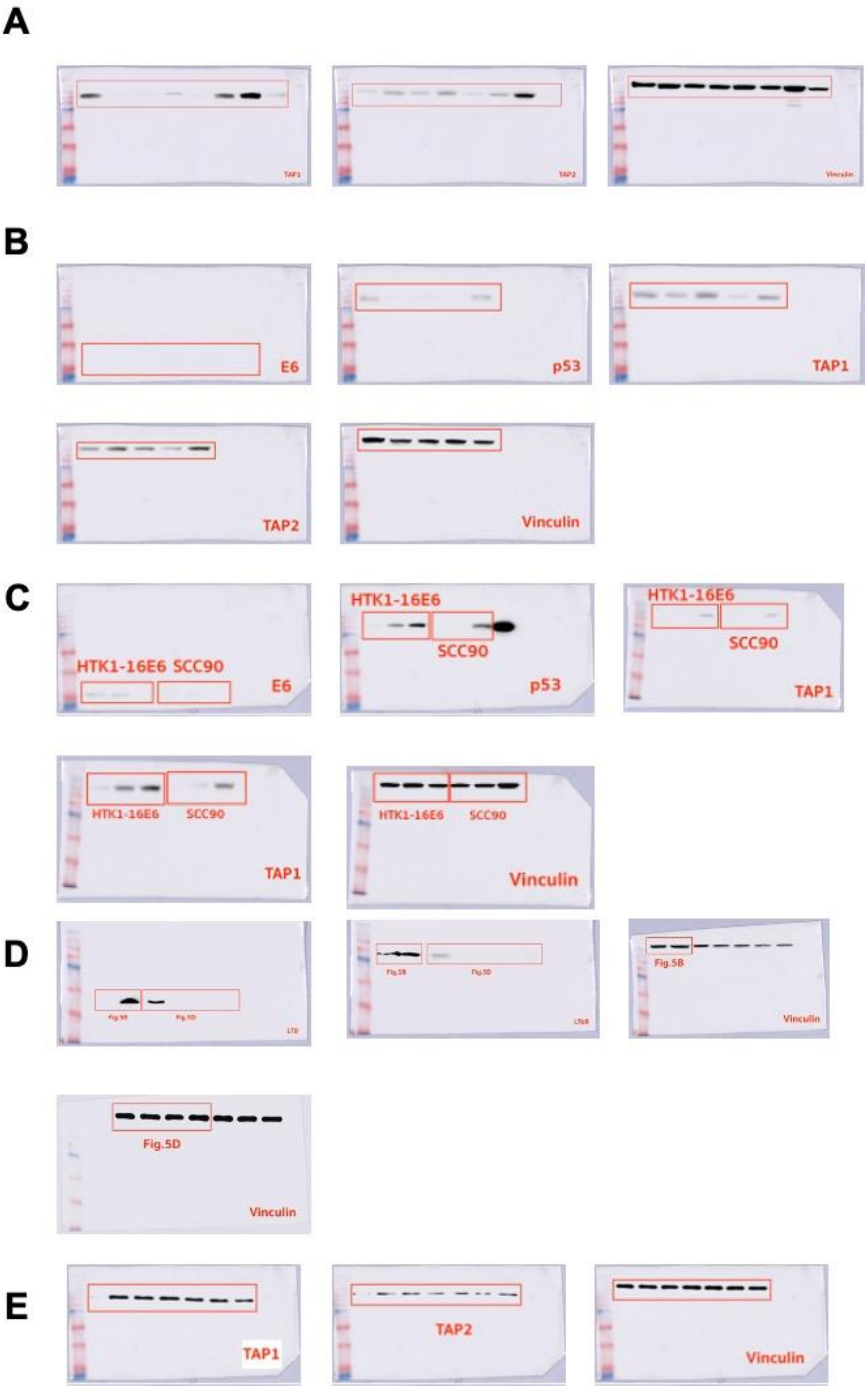

Supplement: Supplementary file 1 [file cancers-14-01944-s001.zip › cancers-1648553-supplementary/Supplementary Figures.pdf]
