# Supplementary material for: Human Papillomavirus 16 E6 Suppresses Transporter Associated with Antigen-Processing Complex in Human Tongue Keratinocyte Cells by Activating Lymphotoxin Pathway
Source: Cancers (Basel). 2022 Apr 12;14(8):1944. doi: 10.3390/cancers14081944 (PMC9028769; doi:10.3390/cancers14081944)
Supplement: Supplementary file 1 [file cancers-14-01944-s001.zip › cancers-1648553-supplementary/Supplementary Tables.pdf]

**Supplementary Table S1** Clinico-demographic information of OSCC specimens.

| Characteristic | Number with each characteristic | HPV positive (%) |
|---|---|---|
| **Gender** | | |
| Male, M | 53 | 29 (54.7%) |
| Female, F | 95 | 55 (57.9%) |
| **Age, year** | | |
| ≤ 50 | 25 | 14 (56.0%) |
| 51-64 | 34 | 18 (52.9%) |
| ≥ 65 | 89 | 52 (58.4%) |
| **Histology grading** | | |
| Well | 97 | 58 (59.8%) |
| Moderately | 38 | 18 (47.4%) |
| Poorly | 5 | 2 (40.0%) |
| Other | 8 | 6 (75.0%) |
| **Anatomical subsite** | | |
| Buccal mucosa | 70 | 39 (55.7%) |
| Tongue | 20 | 17 (85.0%) |
| Lip | 16 | 13 (81.2%) |
| Palate | 18 | 7 (38.8%) |
| Others | 24 | 8 (33.3%) |

**Supplementary Table S2** List of human tongue keratinocytes (HTK) and OSCC cells lines.

| Name | Origin | Refs. |
|---|---|---|
| HTK1 K4DT-pCLXSN-18E6E7 | Human tongue keratinocyte | Zushi et. al., 2011 |
| HTK1 K4DT-pCLXSN-16E6E7 | Human tongue keratinocyte | |
| HTK1 K4DT-pCLXSN-16E6SD | Human tongue keratinocyte | |
| HTK1 K4DT-pCLXSN-16E7 | Human tongue keratinocyte | |
| HTK1 K4DT-pCLXSN | Human tongue keratinocyte | |
| HTK1K4DT-pSI-MSCV-CLXPpuro-H1R-p53Ri | Human tongue keratinocyte | |
| HSC1 | Oral cavity | Kondo et. al., 1981 |
| HSC2 | Oral cavity: floor of mouth | Momose et. al., 1989 |
| HSC3 | Tongue | Momose et. al., 1989 |
| SCCKN | Tongue | Urade et. al., 1992 |
| SCC90 (HPV16$^{+}$ cells) | Tongue | White et. al., 2007 |
| SCC25 | Tongue | Rheinwald et. al., 1981 |
| SCCTH | Oral cavity | White et. al., 2007 |
| UM-SCC-14A | Oral cavity: floor of mouth | Baker et. al., 1985 |

**References:**

1. Zushi, Y., Narisawa-Saito, M., Noguchi, K., Yoshimatsu, Y., Yugawa, T., Egawa, N., Fujita, M., Urade, M., & Kiyono, T. (2011). An in vitro multistep carcinogenesis model for both HPV-positive and -negative human oral squamous cell carcinomas. American journal of cancer research, 1(7), 869–881.

2. Kondo, S., & Aso, K. (1981). Establishment of a cell line of human skin squamous cell carcinoma in vitro. The British journal of dermatology, 105(2), 125–132. https://doi.org/10.1111/j.1365-2133.1981.tb01196.x

3. Momose, F., Araida, T., Negishi, A., Ichijo, H., Shioda, S., & Sasaki, S. (1989). Variant sublines with different metastatic potentials selected in nude mice from human oral squamous cell carcinomas. Journal of oral pathology & medicine : official publication of the International Association of Oral Pathologists and the American Academy of Oral Pathology, 18(7), 391–395. https://doi.org/10.1111/j.1600-0714.1989.tb01570.x

4. Urade, M., Ogura, T., Mima, T., & Matsuya, T. (1992). Establishment of human squamous carcinoma cell lines highly and minimally sensitive to bleomycin and analysis of factors involved in the sensitivity. Cancer, 69(10), 2589–2597. https://doi.org/10.1002/1097-0142(19920515)69:10<2589::aid-cncr2820691032>3.0.co;2-y

5. White, J. S., Weissfeld, J. L., Ragin, C. C., Rossie, K. M., Martin, C. L., Shuster, M., Ishwad, C. S., Law, J. C., Myers, E. N., Johnson, J. T., & Gollin, S. M. (2007). The influence of clinical and demographic risk factors on the establishment of head and neck squamous cell carcinoma cell lines. Oral oncology, 43(7), 701–712. https://doi.org/10.1016/j.oraloncology.2006.09.001

6. Rheinwald, J. G., & Beckett, M. A. (1981). Tumorigenic keratinocyte lines requiring anchorage and fibroblast support cultured from human squamous cell carcinomas. Cancer research, 41(5), 1657–1663.

7. Baker S. R. (1985). An in vivo model for squamous cell carcinoma of the head and neck. The Laryngoscope, 95(1), 43–56. https://doi.org/10.1288/00005537-198501000-00012

**Supplementary Table S3** Oligonucleotides used for PCR and qRT-PCR detection.

| Name | | Sequence (5′→3′) | Amplicon size (bp) |
|---|---|---|---|
| *HPV L1* | GP5+ | TTTGTTACTGTGGTAGATACTAC | 150 |
| | GP6+ | GAAAAATAAACTGTAAATCATATTC | |
| *HPV16E6* | Forward | GTTACTGCGACGTGAGGTATATG | 94 |
| | Reverse | ATTTATCACATACAGCATATGGATTC | |
| *HPV18E6* | Forward | CAGAGGTATTTGAATTTGCATTT | 85 |
| | Reverse | AATCTATACATTTATGGCATGCAG | |
| $LT\alpha_1\beta_2$ | Forward | AGCCACTTCTCTGGTGACCT | 106 |
| | Reverse | GTCGGCCGTCTCCGTTAC | |
| *TAP1* | Forward | CGTCCACCCTGAGTGATTCT | 103 |
| | Reverse | GACACTGATCCCCAGAGCAT | |
| *TAP2* | Forward | AGGAGGCTGCTTCACCTACA | 124 |
| | Reverse | TGAGTTCAGCTCCCCTGTCT | |
| *GAPDH* | Forward | TCATCAGCAATGCCTCCTGCA | 117 |
| | Reverse | TGGGTGGCAGTGATGGCA | |

**Supplementary Table S4** The information of antibodies.

| Targets | Host | React with | REF. |
| --- | --- | --- | --- |
| MHC I W6/32 (HLA IA, B, C) | Mouse monoclonal | Human | Santa Cruz Biotechnology (sc-32235) |
| LT$\alpha_1\beta_2$ | Rabbit polyclonal | Human | Abcam (ab64835) |
| LTβR | Rabbit polyclonal | Human | Abcam (ab70063) |
| TAP1(B-8) | Mouse monoclonal | Human | Santa Cruz Biotechnology (sc-376796) |
| TAP2 (2.17) | Mouse monoclonal | Human | MBL Life science |
| HPV16 E6 (46A4) | Mouse monoclonal | Human | In house production |
| TP53 | Mouse monoclonal | Human | Calbiochem (OP-43) |

**Supplementary Table S5** Oligonucleotide sequences for shRNAs

| Target | Sequence (5′→3′) |
|---|---|
| **shLTβR** | |
| 3913_LTBRRi2_S | GATCCCCGCCCAAGGAACCAATTTATTTCAAGAGAATAAATTGGTTCCTTGGGCTTTTTGGAAA |
| 3914_LTBRRi2_AS | AGCTTTTCCAAAAAGCCCAAGGAACCAATTTATTCTCTTGAAATAAATTGGTTCCTTGGGCGGG |
| 3915_LTBRRi3_S | GATCCCCGCAGGGCACTTCCAGAATATTCAAGAGATATTCTGGAAGTGCCCTGCTTTTTGGAAA |
| 3916_LTBRRi3_AS | AGCTTTTCCAAAAAGCAGGGCACTTCCAGAATATCTCTTGAATATTCTGGAAGTGCCCTGCGGG |
| 3917_LTBRRi4_S | GATCCCCGGCACAGAAGCCGAGGTCACATTCAAGAGATGTGACCTCGGCTTCTGTGCCTTTTTGGAAA |
| 3918_LTBRRi4_AS | AGCTTTTCCAAAAAGGCACAGAAGCCGAGGTCACATCTCTTGAATGTGACCTCGGCTTCTGTGCCGGG |
| **shLT$\alpha_1\beta_2$** | |
| 3919_LTBRi1_S | GATCCCCGGCCTCTATTACCTCTACTTTCAAGAGAAGTAGAGGTAATAGAGGCCTTTTTGGAAA |
| 3920_LTBRi1_AS | AGCTTTTCCAAAAAGGCCTCTATTACCTCTACTTCTCTTGAAAGTAGAGGTAATAGAGGCCGGG |
| 3921_LTBRi2_S | GATCCCCGCCTCTATTACCTCTACTGTTCAAGAGACAGTAGAGGTAATAGAGGCTTTTTGGAAA |
| 3922_LTBRi2_AS | AGCTTTTCCAAAAAGCCTCTATTACCTCTACTGTCTCTTGAACAGTAGAGGTAATAGAGGCGGG |
| 3923_LTBRi3_S | GATCCCCGAGAGGGAAGACCTTCTTTTTCAAGAGAAAAGAAGGTCTTCCCTCTCTTTTTGGAAA |
| 3924_LTBRi3_AS | AGCTTTTCCAAAAAGAGAGGGAAGACCTTCTTTTCTCTTGAAAAAGAAGGTCTTCCCTCTCGGG |
| **siHPV16E6** | |
| #1_16E6 (211-236) _S | GAUAAAUGUUUAAAGUUUUAUUCTA |
| #1_16E6 (211-236) _AS | UAGAAUAAAACUUUAAACAUUUAUCAC |
| #2_16E6 (432-457) _S | GUCUUGUUGCAGAUCAUCAAGAACA |
| #2_16E6 (432-457) _AS | UGUUCUUGAUGAUCUGCAACAAGACAU |
